# Altered cortical thickness-based structural covariance networks in type 2 diabetes mellitus

**DOI:** 10.3389/fnins.2024.1327061

**Published:** 2024-01-24

**Authors:** Yang Huang, Xin Zhang, Miao Cheng, Zhen Yang, Wanting Liu, Kai Ai, Min Tang, Xiaoling Zhang, Xiaoyan Lei, Dongsheng Zhang

**Affiliations:** ^1^Department of MRI, Shaanxi Provincial People’s Hospital, Xi’an, China; ^2^Department of Clinical and Technical Support, Philips Healthcare, Xi’an, China

**Keywords:** type 2 diabetes mellitus, cortical thickness, structural network, topological properties, neuroimaging

## Abstract

Cognitive impairment is a common complication of type 2 diabetes mellitus (T2DM), and early cognitive dysfunction may be associated with abnormal changes in the cerebral cortex. This retrospective study aimed to investigate the cortical thickness-based structural topological network changes in T2DM patients without mild cognitive impairment (MCI). Fifty-six T2DM patients and 59 healthy controls underwent neuropsychological assessments and sagittal 3-dimensional T1-weighted structural magnetic resonance imaging. Then, we combined cortical thickness-based assessments with graph theoretical analysis to explore the abnormalities in structural covariance networks in T2DM patients. Correlation analyses were performed to investigate the relationship between the altered topological parameters and cognitive/clinical variables. T2DM patients exhibited significantly lower clustering coefficient (C) and local efficiency (Elocal) values and showed nodal property disorders in the occipital cortical, inferior temporal, and inferior frontal regions, the precuneus, and the precentral and insular gyri. Moreover, the structural topological network changes in multiple nodes were correlated with the findings of neuropsychological tests in T2DM patients. Thus, while T2DM patients without MCI showed a relatively normal global network, the local topological organization of the structural network was disordered. Moreover, the impaired ventral visual pathway may be involved in the neural mechanism of visual cognitive impairment in T2DM patients. This study enriched the characteristics of gray matter structure changes in early cognitive dysfunction in T2DM patients.

## 1 Introduction

Type 2 diabetes mellitus (T2DM) is a chronic metabolic disease characterized by long-term hyperglycemia and insulin resistance (Chatterjee et al., 2017), and cognitive dysfunction is being increasingly recognized as a common complication and comorbidity of T2DM (Koekkoek et al., 2015). Hyperglycemia and insulin resistance cause a neurodegenerative cascade in the central nervous system by increasing the levels of advanced glycation end-products, oxidative stress, Aβ deposition, and vascular endothelial cell damage, which reduce cognitive reserve (Strachan and Lawrence Lecture, 2011; Dobi et al., 2019). Cognitive impairment is an insidious and progressive process (Nelson et al., 2009), and patients showing mild cognitive impairment (MCI) show a high probability of progression to dementia (Gauthier et al., 2006). Therefore, exploration of the neuroimaging characteristics of early-stage cognitive impairment in T2DM patients may reveal potential clinical therapeutic targets to prevent or delay the occurrence and development of cognitive impairment in T2DM.

Many previous neuroimaging studies have used different methods to explore the patterns of brain structural and functional abnormalities in early cognitive impairment in T2DM patients. Resting-state functional magnetic resonance imaging (fMRI) studies showed that T2DM patients without MCI had extensive abnormal spontaneous neural activity, mainly located in the occipital lobe and temporal regions (Yu et al., 2019; Li et al., 2021). Some studies have also identified disordered functional connectivity (FC) in the hippocampus (Sun et al., 2018) and posterior cingulate cortex (Cheng et al., 2021) regions and the whole brain, and decreased FC in the hippocampus and precuneus has been associated with episodic memory impairment in T2DM patients without MCI (Zhou et al., 2010). Moreover, abnormal changes in brain structure were also observed in T2DM patients without MCI. Diffusion tensor imaging (DTI) has also demonstrated the microstructural abnormalities, and the disruption of white matter tracts connecting the frontal, parietal, and temporal regions was related to slowing of information-processing speed in T2DM patients (Hsu et al., 2012; Reijmer et al., 2013). In addition, multiple studies have shown reduced gray matter volume and cortical thickness in the cingulate gyrus, superior frontal cortex, inferior temporal and middle occipital cortex in T2DM without MCI (Zhang et al., 2014; Li et al., 2018; He et al., 2022). Although these studies have demonstrated extensive structural and functional brain damage and were associated with impairment of multiple cognitive domains before the onset of clinical symptoms of MCI in T2DM patients, their findings mostly reflected abnormal changes within local or regional areas of the brain, and the large-scale whole-brain network change patterns in T2DM without MCI remain poorly understood.

The brain is a complex system, and many neuroimaging studies of neurodegenerative diseases have confirmed that brain abnormalities involve alterations in discrete brain regions and are also characterized by tissue alterations in several functionally and anatomically interrelated regions (Seeley et al., 2009; Raj and Powell, 2018). Graph theory-based analysis methods can serve as a powerful tool to characterize global and local topological properties of brain organization (He and Evans, 2010), which can reflect abnormal changes in brain structural and functional networks. Previous studies have revealed abnormal topological properties of white matter structural networks (Zhang et al., 2019) and disorders of brain functional networks (Chen et al., 2017) in T2DM patients without MCI, and have shown that disorders in topological properties are correlated with the cognitive performance of T2DM patients. However, the changes in gray matter structural network patterns in T2DM patients without MCI and their relationship with cognitive function remain unclear. Compared with the functional and white matter structural covariance network, the gray matter structural covariance network can better reflect the precise coordination of cerebral cortex morphology. Cortical thickness can reflect the density, size and distribution of cells, including neurons, glia and nerve fibers (Kim et al., 2020), and it has high sensitivity in identifying early brain structural changes of gray matter structure (Kunst et al., 2019). Cortical thickness-based network analysis may reflect coordinated changes between underlying neuronal substrates through their anatomical connections (Chen et al., 2008), which could reveal the gray matter alteration features of early-stage cognitive dysfunction in T2DM patients.

To our knowledge, this study is the first to apply a cortical thickness-based method and graph theoretical analysis to explore the abnormalities in structural covariance networks in T2DM patients. We hypothesized that the T2DM patient group would show abnormal network topological organization, and association with some cognitive domains dysfunction before the onset of clinical symptoms of MCI in T2DM patients, which would help characterize the neuropathologic of T2DM-related brain damage.

## 2 Materials and methods

### 2.1 Participants

In this study, 57 hospitalized T2DM patients in the Department of Endocrinology of Shaanxi Provincial People’s Hospital from May 2018 to October 2022 and 60 healthy controls (HCs) matched for age, gender, and education were recruited by advertisement. All participants were aged 45–70 years, right-handed, and had at least 6 years of education, a Mini-Mental State Examination (MMSE) score ≥ 27, and a Montreal Cognitive Assessment (MoCA) score ≥ 26. The inclusion criteria for the HC group were as follows: no symptoms of T2DM; fasting blood glucose (FBG) concentration of <6.1 mmol/L; and glycated hemoglobin (HbA1c) level of <6.0%. T2DM was diagnosed in accordance with the 2014 American Diabetes Association diagnostic criteria: HbA1c ≥ 6.5%; fasting blood glucose ≥7.0 mmol/L; oral glucose tolerance test (OGTT) 2 h postprandial blood glucose ≥11.1 mmol/L; symptoms of hyperglycemia or hyperglycemic crises and random blood glucose ≥11.1 mmol/L, without symptoms of hyperglycemia, and the standard of 1 to 3 items was reviewed, and T2DM patients were receiving stable therapy (diet, oral medications, and/or insulin).

Exclusion criteria for all participants (T2DM and HC) were as follows: severe claustrophobia or contraindications to magnetic resonance imaging (MRI); Parkinson’s disease, alcoholism, major depression, epilepsy, brain injury, or other neurological or psychiatric disorders; or any other systemic disease. In addition, T2DM patients with a history of hypoglycemia (blood glucose concentration < 3.9 mmol/L) or hyperglycemia (blood glucose concentration > 33.3 mmol/L) were also excluded.

Every participant arrived at the department for an MRI scan at 6:30–7:00 p.m. after dinner and underwent blood glucose control in accordance with their doctor’s instructions on the day of the scan. The MRI scans were performed after a structured clinical interview of approximately 30 min and a series of psychological tests. The test procedure and scan time were the same for HCs and T2DM patients. However, to ensure relatively stable blood glucose levels when the participants completed the examination, only one participant underwent scans each day. All participants remained awake during the scan and did not experience discomfort. This study was approved by the ethics committee of Shaanxi Provincial People’s Hospital, and all participants provided written informed consent. All methods were performed in accordance with the relevant guidelines and regulations of the Declaration of Helsinki.

### 2.2 Clinical and neuropsychological test data

The medical history and clinical data of the participants, including blood pressure, height, weight, and body mass index (BMI), were obtained from medical records and questionnaires. In addition, the glycated hemoglobin (HbA1c), fasting blood glucose (FBG), triglyceride (TG), total cholesterol (TC), and low-density lipoprotein cholesterol (LDL-C) levels were measured by standard laboratory testing. A series of neuropsychological tests were used to evaluate the participants’ mental status and cognitive condition. The MMSE and MoCA were used to assess general cognitive function; the Color Trails Test part 1 and part 2 (CTT-1 and CTT-2) were used to test attention and executive functions; the Clock-Drawing Test (CDT) was used to evaluate visuospatial skills; the total immediate recall and delayed recall scores in the Rey Auditory Verbal Learning Test (RAVLT) were used for assessing memory function; and processing speed was evaluated using the Symbol Digit Modalities Test (SDMT). All neuropsychological tests were administered by a psychiatrist with at least 5 years of experience. All participants completed the full battery of neuropsychological tests, except 12 HCs who did not participate in the RAVLT and SDMT.

### 2.3 MRI data acquisition

The MRI scans were acquired using a 3.0 T MR scanner (Ingenia, Philips Healthcare, The Netherlands) with a 16-channel phased-array head coil. Routine T2-weighted and fluid-attenuated inversion recovery (FLAIR) sequences were used to exclude visible brain lesions. The age-related white matter change scale (Wahlund et al., 2001) was used to evaluate lacunar infarcts and white matter hyperintensity based on FLAIR images; participants with scores >2 were excluded. Sagittal three-dimensional T1-weighted imaging (T1WI) scans were obtained using a fast spoiled gradient echo sequence with the following parameters: repetition time (TR) = 7.5 ms, excitation time (TE) = 3.5 ms, flip angle (FA) = 8°, field of view (FOV) = 250 mm × 250 mm, matrix = 256 × 256, slice thickness = 0.55 mm (no gap), and 328 sagittal slices. Scanning range covers the whole brain.

### 2.4 Processing of MRI data

T1-weighted structural images were processed using the Computational Anatomy Toolbox 12 (CAT12)^1^ software for SPM12 in MATLAB. Image preprocessing included correction for bias-field inhomogeneities; tissue segmentation into gray matter (GM), white matter (WM), and cerebrospinal fluid (CSF); affine registration to an MNI template space; and subsequent nonlinear deformation. Subsequently, cortical thickness (CT) estimation was performed automatically in CAT12 using projection-based thickness (PBT) method (Dahnke et al., 2013). On the basis of the Destrieux atlas (Destrieux et al., 2010), the surface was parcellated into 148 cortical regions (74 in each hemisphere), and the cortical thickness of the 148 cortical regions was extracted using CAT12. Finally, only participants with a weighted average score of B+ or higher in the quality report of CAT12 were included in further analysis.

### 2.5 Construction and analysis of structural covariance networks

To investigate the cortical thickness-based structural covariance network alterations in T2DM patients, we applied graph theoretical methods using the Graph Analysis Toolbox (GAT; Hosseini et al., 2012). For each participant, a 148 × 148 correlation matrix was constructed by calculating Pearson correlation coefficients between cortical thickness values of each cortical region, with age, sex, and total intracranial volume (TIV) as covariates (He et al., 2007; Bernhardt et al., 2011). For each correlation matrix, a binary matrix was derived after thresholding, and the correlation coefficient was considered to be 1 if it was above the threshold, otherwise the correlation coefficient was 0 (He et al., 2008). To ensure that T2DM patients and HC networks had the same number of nodes and edges at each density, these thresholds were defined as a range of network densities from 0.1 to 0.4 (step = 0.01) in this study. When the density was below 0.1, the number of edges was less than the number of nodes and the network was considered a fragmented network, while when the density above 0.5, the network showed a nearly random configuration (Humphries et al., 2006; Singh et al., 2013).

A series of global and regional network metrics were used to characterize the topological properties of the cortical thickness-based structural covariance networks. The network metrics included clustering coefficient (C) and characteristic path length (Lp). The network metrics C and Lp were normalized by comparable values of 1,000 random networks to obtain the normalized clustering coefficient (gamma = C real/C rand >1), normalized characteristic path length (lambda = Lp real/Lp rand ≈ 1), and the ratio of gamma and lambda was defined as the small-worldness (sigma = gamma/lambda >1). Additionally, we calculated the global efficiency (Eglobal) and the local efficiency (Elocal) parameters to measure the network efficiency of transmitting information at the global and local levels. Furthermore, the nodal metrics of structural network including betweenness centrality (BC), degree centrality (DC), and nodal efficiency (NE) were calculated in each individual node of the structural network. DC reflects the importance of the node or brain region in the whole-brain network, while BC characterizes the ability of the node to influence the entire network. In addition, NE represents the efficiency of the parallel information transmission capability of nodes in the structural network. Finally, the area under the curve (AUC) was calculated for each network metric to provide a scalar that does not depend on specific threshold selection (He et al., 2009; Wang et al., 2009).

### 2.6 Statistical analyses

Statistical analyses were performed using SPSS Statistics version 24 (IBM Corporation, Armonk, NY, United States). The independent-sample *t*-test was used to detect group differences for normally distributed variables, while the Mann–Whitney *U*-test was used for non-normally distributed variables. The Chi-square test (*χ*^2^) was used to assess group differences in sex. The significance level was set at *p* < 0.05.

To test the statistical significance of the differences between groups in network topological metrics, non-parametric permutation tests with 1,000 repetitions were performed in GAT. Along with the permutation tests, AUC analysis was performed to assess the between-group differences across all network densities (Zhang et al., 2019). The significance level was set at *p* < 0.05 for group differences in global network metrics, and the statistical threshold was set at *p* < 0.05 (FDR-corrected) for the nodal network metrics.

**Figure 1:**
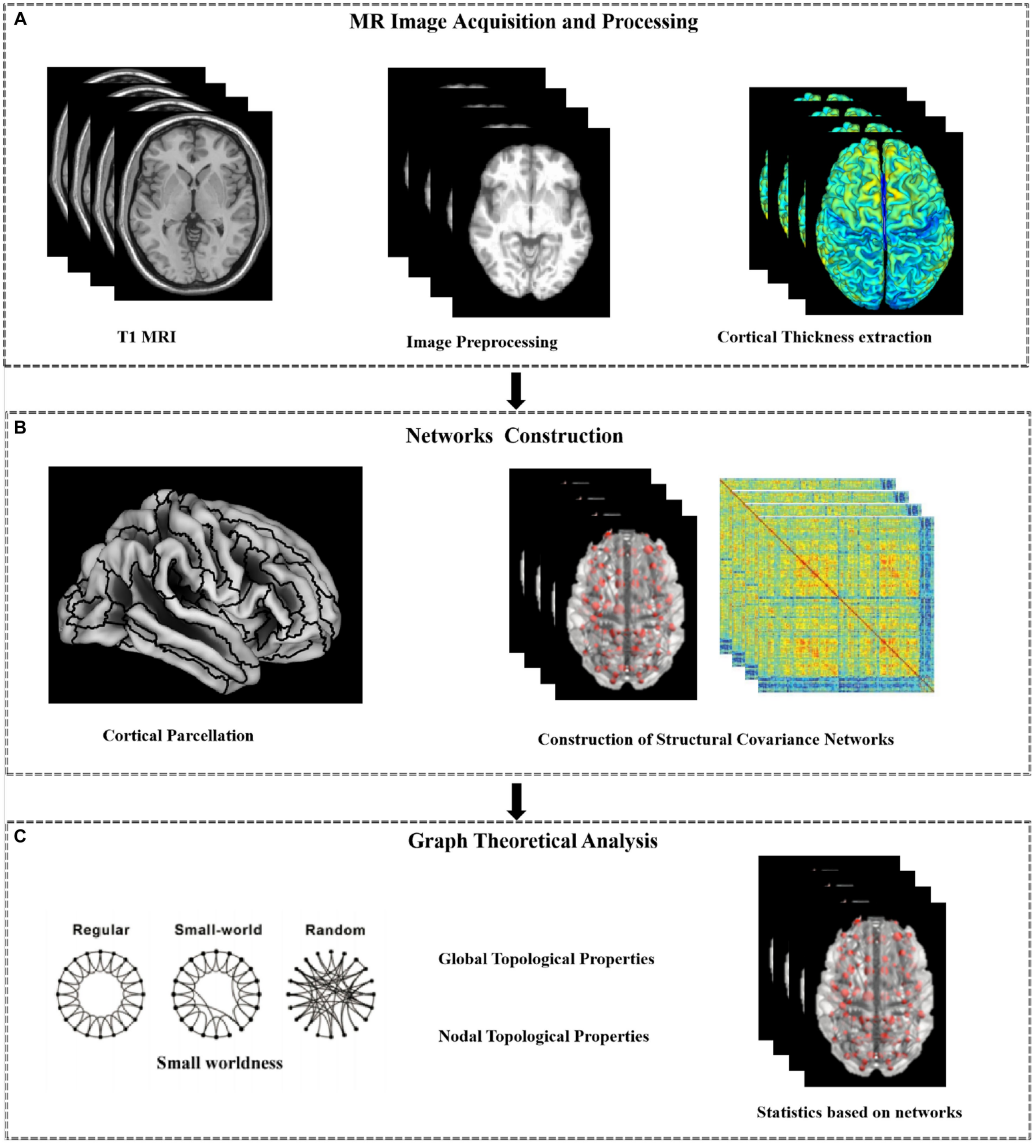
Work flow of the analysis. The proposed method consists of three steps: MR Image Acquisition and Preprocessing **(A)**; Networks Construction**(B)**; Graph Theoretical Analysis **(C)**.

To investigate the correlations between topological properties of the structural network and clinical/cognitive variables, partial correlation (Bonferroni corrected, *p* < 0.05) was further performed in T2DM patient group, with age, gender, BMI, and TIV values as covariates (Figure 1).

## 3 Results

### 3.1 Clinical and neuropsychological data

One T2DM patient showing excessive motion and one healthy control with quality report score lower than B+ were excluded from the study. A total of 56 patients with T2DM and 59 HCs were enrolled in the study. According to previous studies to evaluate the stability of neuroimaging (Grady et al., 2021; Helwegen et al., 2023), in this study, the effect size of the current sample size was 0.56 and the power was 0.85, which indicated that the results of this study were highly reliable. The demographic, clinical, and neuropsychological data of the two groups were presented in Table 1. The two groups showed no significant differences in age, sex, education level, blood pressure, TG concentration, TC concentration, LDL-C concentration, and CTT-2, MMSE, MoCA, CDT, RAVLT immediate/delay, and SDMT scores (*p* > 0.05). However, the BMI and CTT-1 score were higher in the T2DM group (all *p* < 0.05). The T2DM group showed higher levels of FBG and HbA1c than the control group (all *p* < 0.001). The T2DM group included 27 patients with no complications and 29 patients with complications such as nephropathy, peripheral neuropathy, and retinopathy (Supplementary Table S1).

**Table 1:** Demographic, clinical, and neuropsychological characteristics of the participants.

Variable	T2DM (*n* = 56)	HC (*n* = 59)	*t*/*χ*^2^ value	Value of *p*
Sex (male/female)	42/14	37/22	3.042	0.081^#^
Age (years)	52.89 ± 10.28	54.05 ± 5.94	−0.902	0.362
Education (years)	14.32 ± 2.24	13.90 ± 3.53	0.858	0.382
Duration (years)	7.37 ± 5.69	–	–	0
Systolic BP (mmHg)	125.92 ± 16.93	123.07 ± 11.02	0.822	0.414
Diastolic BP (mmHg)	79.42 ± 10.20	79.51 ± 8.87	−0.050	0.955
BMI (kg/m^2^)	27.63 ± 9.00	25.93 ± 3.77	2.433	0.021^*^
FBG (mmol/L)	8.32 ± 3.13	4.10 ± 2.06	8.741	<0.001^*^
HbA1c (%)	8.07 ± 2.04	5.32 ± 1.21	8.884	<0.001^*^
TG (mmol/L)	2.43 ± 1.63	1.81 ± 1.04	1.639	0.100
TC (mmol/L)	4.33 ± 1.44	4.30 ± 1.17	0.123	0.903
LDL (mmol/L)	2.62 ± 0.90	2.65 ± 0.82	−0.191	0.849
CTT-1	74.98 ± 25.54	66.03 ± 16.17	2.371	0.023*
CTT-2	151.89 ± 48.47	136.29 ± 55.83	1.632	0.105
MMSE	29.05 ± 0.94	28.86 ± 1.52	0.788	0.432
MoCA	27.41 ± 1.35	27.60 ± 1.21	−0.828	0.409
CDT	27.16 ± 2.18	28.95 ± 2.94	−1.635	0.103
RAVLT immediate	43.34 ± 6.70	44.85 ± 8.63	−0.788	0.433
RAVLT delay	8.38 ± 24.83	9.14 ± 3.07	−1.064	0.290
SDMT	45.72 ± 12.97	48.19 ± 7.01	−0.907	0.367

Normally distributed variables are presented as mean ± standard deviation, and variables that are non-normally distributed are presented as median (minimum, maximum). T2DM, type 2 diabetes mellitus; HC, healthy control; BMI, body mass index; FBG, fasting blood glucose; HbA1c, glycated hemoglobin; TG, triglyceride; TC, total cholesterol; LDL-C, low-density lipoprotein cholesterol; CTT-1, Color Trails Test part 1; CTT-2, Color Trails Test part 2; MMSE, Mini-Mental State Examination; MoCA, Montreal Cognitive Assessment; CDT, Clock-Drawing Test; RAVLT, Rey Auditory Verbal Learning Test; SDMT, Symbol Digit Modalities Test. ^#^*p* for the *χ*^2^ test; ^*^p < 0.05.

### 3.2 Global topological properties

**Figure 2:**
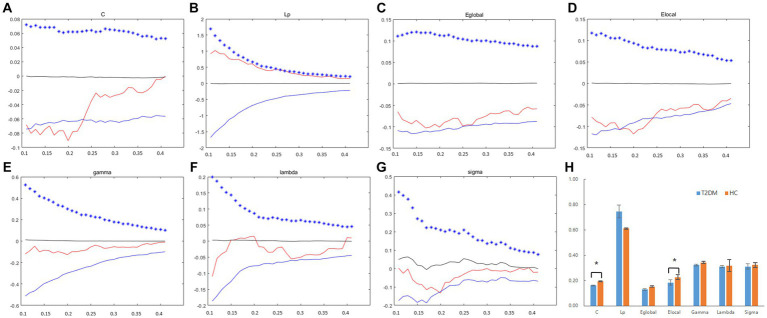
The differences in global topological metrics in the structural network between the T2DM and HC groups. Graphs **(A–G)** show the small-world properties and network efficiency under different sparsity values (0.1 < S < 0.4). The AUC of small-world properties and network efficiency over the full sparsity range is shown by histogram graphs **(H)**. The symbol “*” denotes statistical significance. C, clustering coefficient; Lp, characteristic path length; Eglobal, global efficiency; Elocal, local efficiency; gamma, normalized clustering coefficient; lambda, normalized shortest path length; sigma, small-worldness; AUC, area under the curve.

The data for the global topological metrics, including clustering coefficient (C), characteristic path length (Lp), normalized clustering coefficient (gamma), normalized characteristic path length (lambda), small-worldness (sigma), global efficiency (Eglobal), and local efficiency (Elocal), in each density threshold range of the two groups are presented in Figures 2A–G. Over the defined density range (0.1 < sparsity <0.4, step = 0.01), the differences in the AUCs of the global network properties (C, Lp, gamma, lambda, sigma, Eglobal, Elocal) between the two groups are shown in Figure 2H. The two groups showed no significant differences in Lp, gamma, lambda, sigma, and Eglobal metrics. However, the T2DM group exhibited a significantly lower clustering coefficient (C) and local efficiency (Elocal).

### 3.3 Nodal topological properties

**Figure 3:**
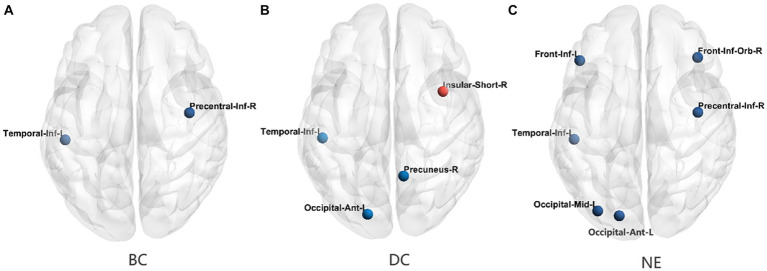
The differences in the node topological properties showing significant differences between the T2DM and HC groups. **(A)** The distribution of the significant BC between the T2DM and HC groups. **(B)** The distribution of the significant DC between the T2DM and HC groups. **(C)** The distribution of the significant NE between the T2DM and HC groups. BC, betweenness centrality; DC, degree centrality; NE, nodal efficiency; Red color indicates increased nodal properties, and the blue color indicates decreased nodal properties.

In comparison with the HCs, the T2DM patients showed decreased BC values in the right inferior precentral sulcus and left inferior temporal gyrus, after FDR correction for *P*. Moreover, the T2DM patients showed decreased DC values in the right precuneus gyrus, left anterior occipital sulcus, and inferior temporal gyrus, and increased DC values in the right short insular gyrus. Additionally, the T2DM patients showed decreased NE values in the right inferior orbital frontal gyrus, inferior precentral sulcus, left middle occipital gyrus, inferior frontal gyrus, and anterior occipital sulcus, and inferior temporal gyrus. The distributions of node properties showing significant differences between groups are presented in Figure 3 and Table 2.

**Table 2:** The significant differences in nodal topological metrics.

Brain regions	BC	DC	NE
Precentral-Inf-R	*P* < 0.001 *T* = −4.059	–	*p* < 0.001 *T* = −3.364
Frontal-Inf-L	–	–	*P* = 0.001 *T* = −3.301
Frontal-Inf-Orbital-R	–	–	*P* < 0.001 *T* = −3.733
Precuneus-R	–	*P* < 0.001 *T* = −3.611	–
Insular-Short-R	–	*P* < 0.001 *T* = 3.428	–
Temporal-Inf-L	*P* = 0.001 *T* = −3.115	*P* < 0.001 *T* = −3.823	*P* < 0.001 *T* = −3.901
Occipital-Ant-L	–	*P* < 0.001 *T* = −3.693	*P* < 0.001 *T* = −3.845
Occipital-Middle-L	–	–	*P* < 0.001 *T* = −3.372

BC, betweenness centrality; DC, degree centrality; NE, nodal efficiency.

### 3.4 Correlation analysis

**Figure 4:**
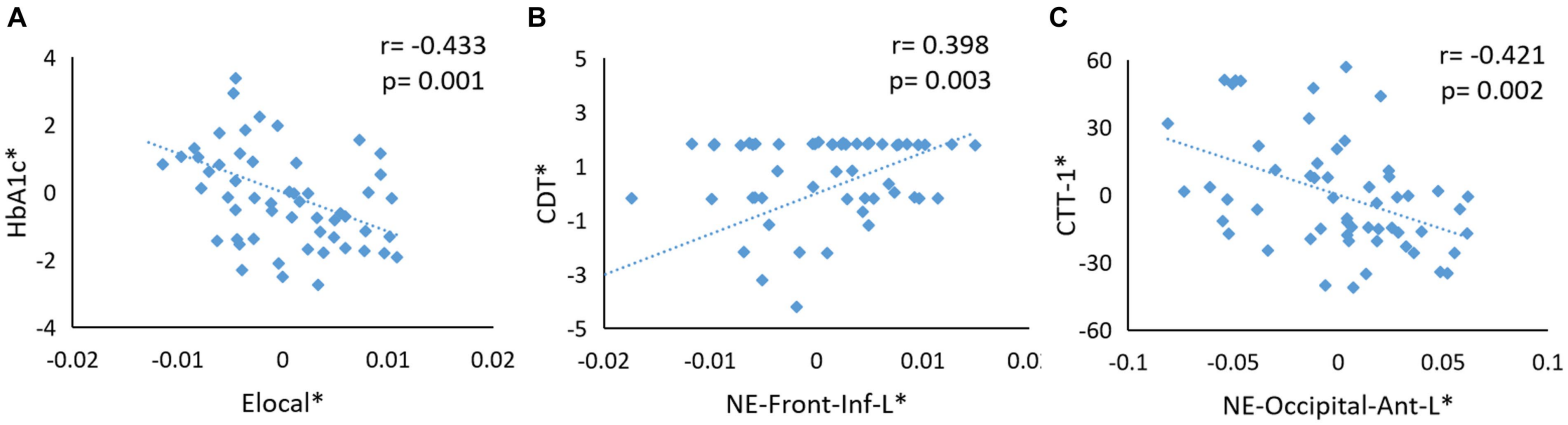
**(A)** The Elocal metrics were negatively correlated with HbA1c level (*p* = 0.001, *r* = −0.433). **(B)** The NE values in the left frontal gyrus were positively related with CDT score (*p* = 0.003, *r* = 0.398). **(C)** The NE values in left anterior occipital sulcus were negatively correlated with CTT-1 score (*p* = 0.002, *r* = −0.421). The asterisk (∗) indicated coordinate values controlling for the influence of gender, age, BMI, and TIV values.

In the T2DM group, the Elocal metrics were negatively correlated with HbA1c level (*p* = 0.001, *r* = −0.433; Figure 4A). Furthermore, the NE values in the left frontal gyrus were positively related to the CDT score (*p* = 0.003, *r* = 0.398; Figure 4B), and the NE values in the left anterior occipital sulcus were negatively correlated with the CTT-1 score (*p* = 0.002, *r* = −0.421; Figure 4C).

## 4 Discussion

Different from previous studies that only focused on the gray matter morphology of T2DM without MCI, this study revealed the changes of gray matter network efficiency and node properties at the large-scale network level. The results showed that there was no significant difference in the global efficiency and small-world properties between T2DM patients and HCs, but we found abnormalities in the local topological organization of the structural network and disorders of multiple node topology properties in T2DM patients. These results indicated that although the global network of T2DM patients without MCI was relatively normal, their local network efficiency may have declined to some extent.

### 4.1 Altered global properties in cortical structural networks in T2DM

The brain is a complex network connected by anatomical tracts or functional associations, and the topology of brain networks shows small-world characteristics (Reijneveld et al., 2007; Bullmore and Sporns, 2009). In this study, although the brain structural network still showed small-world topological properties, patients with T2DM showed lower Cp values in the sparsity range, which may indicate fewer connections between neighboring regions in the network and reduced processing efficiency of local information is reduced in these patients.

The Elocal value represents the efficiency of information exchange between neighboring regions (Rubinov and Sporns, 2010), and previous studies have shown decreased Elocal values in WM networks in T2DM patients (Zhang et al., 2016; Xiong et al., 2022). Our study indicated decreased local efficiency of the gray matter structural network in T2DM patients, which is significantly correlated to the HbA1c level. Thus, the local network efficiency in T2DM patients may be affected by blood glucose levels. Long-term hyperglycemia has been suggested to affect the hub regions of brain networks through the effects of oxidative stress, resulting in reducing efficiency of the brain networks in T2DM (Kim et al., 2016). However, no significant reduction in global network efficiency was observed in T2DM patients without MCI. Thus, the early-stage cognitive impairment of T2DM patients may be relatively mild, and a certain compensatory effect in the local network may maintain the global network efficiency at a relatively normal level.

### 4.2 Altered nodal properties in cortical structural networks in T2DM

The occipital visual network is one of the most vulnerable brain regions in T2DM patients (Macpherson et al., 2017), and multiple previous studies have confirmed extensive impairment of functional (Qin et al., 2019; Zhang et al., 2020) and structural (Zhang et al., 2019) networks of the visual area in T2DM patients. The ventral visual pathway is considered to be the core area of object recognition and perception, processing features in color, shape, and structure of objects (Ungerleider and Haxby, 1994). This pathway transmits visual information from the retina to the primary visual cortex, and then projects them through the inferior temporal to the frontal cortex, including the inferior frontal gyrus (Shen et al., 1999; Kravitz et al., 2013). In this study, the ventral visual pathway regions (occipital cortical, inferior temporal gyrus, inferior frontal gyrus) showed significant reductions in multiple structural topological properties, with the most significant changes appearing in the inferior temporal gyrus. In addition, NE values in the anterior occipital cortex were correlated with the CTT-1 score of T2DM patients, and the reduction in the node efficiency in the IFG was correlated with the CDT score. CTT-1 and CDT are particularly useful tools for detecting changes in visuospatial ability (Freund et al., 2005; Vaportzis et al., 2019), further suggesting that impaired local efficiency of the occipital cortex and IFG may be involved in visuospatial impairment in T2DM patients. The inferior temporal gyrus (ITG) is engaged in visual cognitive processes and involved in the formation of high-level visual representations (Bokde et al., 2006; DiCarlo et al., 2012). Previous studies have reported decreased DC and node efficiency in ITG (Chen et al., 2017), and cortical atrophy in the ITG has been correlated with cognitive decline in T2DM patients (Li et al., 2020). The inferior frontal junction plays a key role in controlling the spatially global effects of feature-based attention in the human visual area (Zhang et al., 2018), and previous studies have shown abnormal neuronal activity (Zhang et al., 2022) and decreased gray matter volume (He et al., 2022) in the IFG regions in T2DM patients. Therefore, we speculate that impaired ventral visual pathways may be involved in the neural basis of visual cognitive impairment in T2DM patients, particularly visuospatial abnormalities. Although the CDT groups showed no statistically significant differences in this study, patients with T2DM tended to show impaired visuospatial function. Future studies using more comprehensive visuospatial scores scales may help to detect possible visuospatial dysfunction in the early stages of T2DM.

Functional impairments (represented by DC values) and structural impairments of the precuneus region have been widely reported in T2DM patients (Chen et al., 2015, 2017; Kang et al., 2022). The precuneus is a highly connected network hub region and an important part of the default mode network, and is involved in many high-order complex cognitive processes (Cavanna and Trimble, 2006). This region is one of the brain areas prone to amyloid deposition. Insulin resistance and hyperglycemia in T2DM patients also accelerate amyloid deposition (Mormino et al., 2011), which may be the reason for the reduced properties of the structural nodes. The precentral region is a part of the sensorimotor network, and the BC and NE values in the precentral region are reduced in T2DM patients, which may indicate sensorimotor abnormalities. Previous studies have demonstrated reduced gray matter volume (Zhang et al., 2020) and cortical thickness (Selvarajah et al., 2019) in the precentral region in patients with diabetic neuropathy. A small-world network analysis showed decreased nodal importance in the precentral gyrus in patients with diabetic neuropathy (Yuan et al., 2022), suggesting that diabetic neuropathy is closely related to abnormal central sensorimotor function. In this study, the T2DM group included 27 patients with peripheral neuropathy, which may have been responsible for the abnormal structural topological properties in the precentral region. However, the presence of central sensorimotor abnormalities in T2DM patients prior to the onset of peripheral neuropathy should be explored in future studies.

The anterior insula is associated with diverse brain networks and plays a role in multiple cognitive functions (Menon and Uddin, 2010). Previous studies have shown that in patients with pre-clinical AD, the increased FC between the anterior insula and the frontal and temporal lobe regions can be considered to be compensatory activity to regulate early functional loss in patients (Wang et al., 2021), and Cui et al. found increased DC in the anterior insula in T2DM patients (Cui et al., 2016). Therefore, we speculate that the increased DC in the right anterior insula in this study may represent a compensatory mechanism for early cognitive impairment in T2DM patients, which may also be one of the reasons for the absence of significant differences in global efficiency in this study.

### 4.3 Limitations

This study had several notable limitations. First, this study only discussed the change characteristics of the cortical thickness covariance network in early stages of T2DM-related cognitive impairment, and we will further observe the brain functional changes in T2DM patients without MCI in the future. Second, the missing RAVLT and SDMT scale scores for some HCs prevented an accurate assessment of the presence of impairments in memory and attention function in T2DM patients. Finally, the differences in the treatment regimens and drugs for T2DM among patients may have introduced some bias, but such differences are difficult to avoid.

## 5 Conclusion

This study found that while the patients showed a relatively normal global network, the topological organization of the local structural network showed abnormalities, and that the disordered topological properties of some nodes in the ventral visual pathway may be involved in the neural basis of visual cognitive impairment in T2DM patients. This study made up for the lack of previous gray matter structure network research in T2DM patients without MCI, which enriched the characteristics of gray matter structure changes in early cognitive dysfunction in T2DM patients, and provided more evidence for elucidating the neural mechanism of T2DM-related cognitive impairment.

## Data availability statement

The original contributions presented in the study are included in the article/Supplementary material, further inquiries can be directed to the corresponding authors.

## Ethics statement

The studies involving humans were approved by the ethics committee of Shaanxi Provincial People’s Hospital. The studies were conducted in accordance with the local legislation and institutional requirements. The participants provided their written informed consent to participate in this study.

## Author contributions

YH: Writing – original draft. XinZ: Data curation. MC: Data curation. ZY: Data curation. WL: Data curation. KA: Methodology. MT: Supervizion. XiaZ: Funding acquisition, Project administration. XL: Project administration. DZ: Writing – review & editing.

## Footnotes

^1^ http://www.neuro.uni-jena.de/cat/
