# Supplementary material for: Altered cortical thickness-based structural covariance networks in type 2 diabetes mellitus
Source: Front Neurosci. 2024 Jan 24;18:1327061. doi: 10.3389/fnins.2024.1327061 (PMC10851426; doi:10.3389/fnins.2024.1327061)
Supplement: Supplementary file 1 [file Table_1.docx]

**Table S1** Types T2DM patients with microvascular complications

| complications | Diabetic Retinopathy (DR) | Diabetic Peripheral Neuropathy (DPN) | Diabetic Nephropathy (DN) |
| --- | --- | --- | --- |
| patient1 | 0 | 0 | 0 |
| patient2 | 0 | 0 | 0 |
| patient3 | 0 | 0 | 1 |
| patient4 | 0 | 0 | 0 |
| patient5 | 0 | 0 | 0 |
| patient6 | 0 | 1 | 1 |
| patient7 | 0 | 1 | 1 |
| patient8 | 0 | 0 | 0 |
| patient9 | 0 | 0 | 1 |
| patient10 | 0 | 0 | 0 |
| patient11 | 0 | 0 | 0 |
| patient12 | 0 | 1 | 0 |
| patient13 | 0 | 1 | 0 |
| patient14 | 0 | 1 | 0 |
| patient15 | 0 | 1 | 0 |
| patient16 | 0 | 1 | 0 |
| patient17 | 0 | 0 | 0 |
| patient18 | 0 | 0 | 0 |
| patient19 | 0 | 0 | 0 |
| patient20 | 0 | 0 | 0 |
| patient21 | 0 | 0 | 0 |
| patient22 | 0 | 1 | 0 |
| patient23 | 0 | 1 | 0 |
| patient24 | 0 | 1 | 0 |
| patient25 | 0 | 0 | 0 |
| patient26 | 0 | 1 | 0 |
| patient27 | 0 | 0 | 0 |
| patient28 | 0 | 0 | 0 |
| patient29 | 1 | 1 | 0 |
| patient30 | 0 | 0 | 0 |
| patient31 | 0 | 0 | 0 |
| patient32 | 0 | 1 | 0 |
| patient33 | 0 | 1 | 0 |
| patient34 | 0 | 0 | 0 |
| patient35 | 0 | 0 | 0 |
| patient36 | 0 | 1 | 0 |
| patient37 | 0 | 0 | 0 |
| patient38 | 0 | 1 | 0 |
| patient39 | 0 | 1 | 0 |
| patient40 | 0 | 0 | 0 |
| patient41 | 0 | 1 | 0 |
| patient42 | 0 | 1 | 0 |
| patient43 | 0 | 0 | 0 |
| patient44 | 0 | 1 | 0 |
| patient45 | 0 | 1 | 0 |
| patient46 | 0 | 0 | 0 |
| patient47 | 0 | 1 | 1 |
| patient48 | 0 | 0 | 0 |
| patient49 | 0 | 1 | 0 |
| patient50 | 0 | 0 | 0 |
| patient51 | 0 | 1 | 0 |
| patient52 | 1 | 1 | 1 |
| patient53 | 0 | 0 | 0 |
| patient54 | 0 | 1 | 0 |
| patient55 | 0 | 0 | 0 |
| patient56 | 0 | 1 | 0 |
